# Phytoplankton Cell Size Reduction in Response to Warming Mediated by Nutrient Limitation

**DOI:** 10.1371/journal.pone.0071528

**Published:** 2013-09-05

**Authors:** Kalista Higini Peter, Ulrich Sommer

**Affiliations:** GEOMAR Helmholtz Centre for Ocean Research Kiel, Kiel, Germany; University of British Columbia, Canada

## Abstract

Shrinking of body size has been proposed as one of the universal responses of organisms to global climate warming. Using phytoplankton as an experimental model system has supported the negative effect of warming on body-size, but it remains controversial whether the size reduction under increasing temperatures is a direct temperature effect or an indirect effect mediated over changes in size selective grazing or enhanced nutrient limitation which should favor smaller cell-sizes. Here we present an experiment with a factorial combination of temperature and nutrient stress which shows that most of the temperature effects on phytoplankton cell size are mediated via nutrient stress. This was found both for community mean cell size and for the cell sizes of most species analyzed. At the highest level of nutrient stress, community mean cell size decreased by 46% per °C, while it decreased only by 4.7% at the lowest level of nutrient stress. Individual species showed qualitatively the same trend, but shrinkage per °C was smaller. Overall, our results support the hypothesis that temperature effects on cell size are to a great extent mediated by nutrient limitation. This effect is expected to be exacerbated under field conditions, where higher temperatures of the surface waters reduce the vertical nutrient transport.

## Introduction

Shrinking of body size has been proposed as one of the universal responses of organisms to global climate warming [1], [2] and related to classic biogeographic rules [3], [4] and to the temperature-size rule (TSR) [5]. Smaller body sizes in warmer climates have been the domain of biogeographic rules since more than 1½ centuries [3], [4]. More recently, interest in the temperature response to size has been revived by Global Change research and by the “metabolic theory of ecology” [6], [7] and phytoplankton has become one of the model systems to study the size effect of warming. While most phytoplankton studies support the general trend [8], [9], [10], the mechanism remain still unresolved. A meta-analysis of monoculture studies with protists found on average a 2.5% shrinkage per °C [5], which is far less than the size trends observed in-situ and in experiments with naturally mixed plankton communities. Besides direct temperature effects, also enhanced size-selective grazing under warmer conditions [8], [9], [11], [12], [13], [14] has been suggested as proximate cause, but it is general knowledge in biological oceanography that small phytoplankton tend to dominate in warm, nutrient poor waters while large ones tend to dominate in cold, nutrient rich waters [15], [16], [17], [18]. However, identification of the causal mechanism is difficult from field data because of the global anti-correlation between temperature and nutrients in the ocean [19]. Warming of the surface waters intensifies vertical density stratification and, thereby, reduces vertical nutrient transport through the thermocline into the well illuminated surface zone.

In order to disentangle nutrient from temperature effects on phytoplankton cell size, we performed an experiment with a factorial combination of temperature and nutrient stress. We subjected mixed plankton assemblages from Kiel Bight, western Baltic Sea, to three temperature levels and three levels of nutrient limitation in a fully factorial design. The levels of nutrient limitation were manipulated by semi-continuous dilution of the cultures three times per week with fresh media and assessed by measuring the particulate matter C∶N ratio, which is the inverse of the carbon-normalized N-cell quota [20] and shows a linear relationship to the extent of nutrient limitation [21].

## Materials and Methods

The field samples taken for our experiment did not involve protected species and were not taken from a protected area. No permit was needed.

The experiment was conducted for three weeks from 9^th^ to 30^th^ August 2012. Twenty seven Erlenmeyer flasks of 700 mL incubated in temperature (13.5, 16.5, and 19.5°C; i.e. in situ conditions and 3°C below and above) and light controlled climate cabinets served as experimental units. They were filled with Baltic Sea water (Kiel Fjord) from 1 to 3 m depth containing the natural plankton community and sieved through plankton gauze of 200 µm mesh size in order to keep out large zooplankton. Microscopic inspection of the initial plankton community indicated that microzooplankton were extremely rare. After measuring the in situ nutrient concentrations, the water was supplemented with nutrients to reach starting concentrations of concentrations of 32.7 µmolL^−1^ NO_3_, 4.47 µmolL^−1^ PO_4_ and 29 Si, µmolL^−1^, respectively. Part of the water was sterile filtered (0.2 µm pore size) and stored in darkness at 2°C to serve a medium for the dilutions of cultures. The strength of nutrient limitation was manipulated by semi continuous dilution three times per week on Monday, Wednesday and Friday in which 0% (N3, strongest nutrient stress), 25% (N2, medium nutrient stress), and 50% (N1, weakest nutrient stress) of the culture volume were replaced by fresh medium.

Samples were taken at the end of experiment. Samples for the elemental analysis (C, N, P) of the particulate matter were filtered onto pre-combusted Whatman GF/F filters (Whatman GmbH, Dassel, Germany). N and C were measured by gas chromatography [22] and P was measured colorimetrically after converting organic phosphorus compounds to orthophosphate [23]. Samples for counting and sizing phytoplankton >5 µm were fixed with Lugol's iodine and analyzed with the inverted microscope methodology [24]. We counted at least 100 individuals per species to achieve 95% confidence limits of ca, ±20%. Cell size measurements were taken from 20 randomly chosen individuals per species and per experimental unit and volumes were calculated after approximation to geometric proxies [25]. Phytoplankton <5 µm were counted and sized by flow cytometry. Two size metrics were used to assess the response to the experimental treatments: the cell volume of individual phytoplankton species (*Vi*) and the mean cell volume of the phytoplankton community (*Vc*) which was obtained by dividing total volume by the total cell number.

## Results

**Figure 1:**
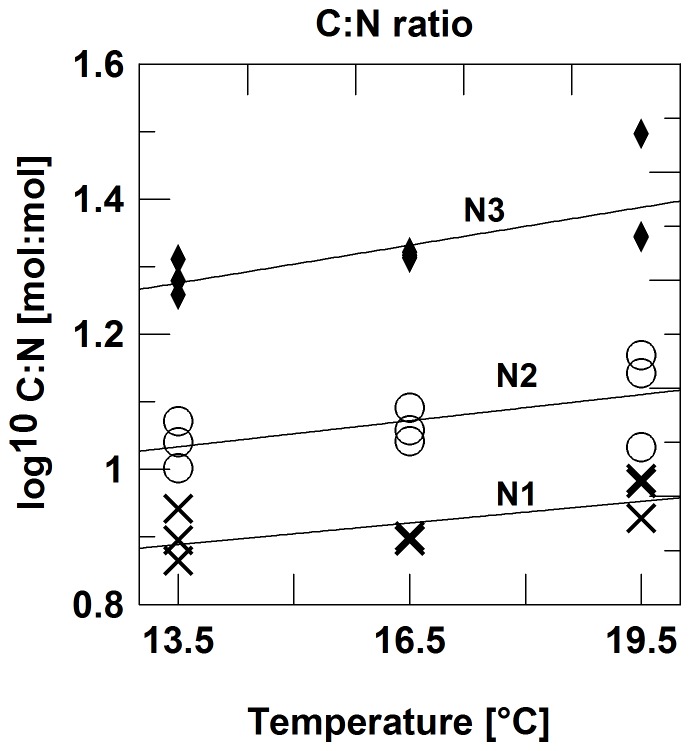
Particular matter C∶N ratios increase with tmeperature and decrease with dilution rate. Molar C∶N ratios of particulate, organic matter in response (log^10^-scale) to temperature and nutrient regime; N1: 50% dilution three times per week; N2: 25% dilution three times per week; N3: no dilution.

The C∶N ratios of particulate matter increased in the direction N1 to N3 and with temperature (Fig. 1). A two-factor ANOVA with log-transformed C∶N data showed a significant main effect of nutrient treatment and of temperature, but no interaction effect (*P_nutr_*<0.0001; *P_temp_* = 0.0031; *N* = 27). The molar C∶N ratios ranged from 8.5 to 37, thus indicating weak to severe nitrogen limitation [21] while P-limitation could be excluded because of N∶P ratios <16 in all experimental units.

**Figure 2:**
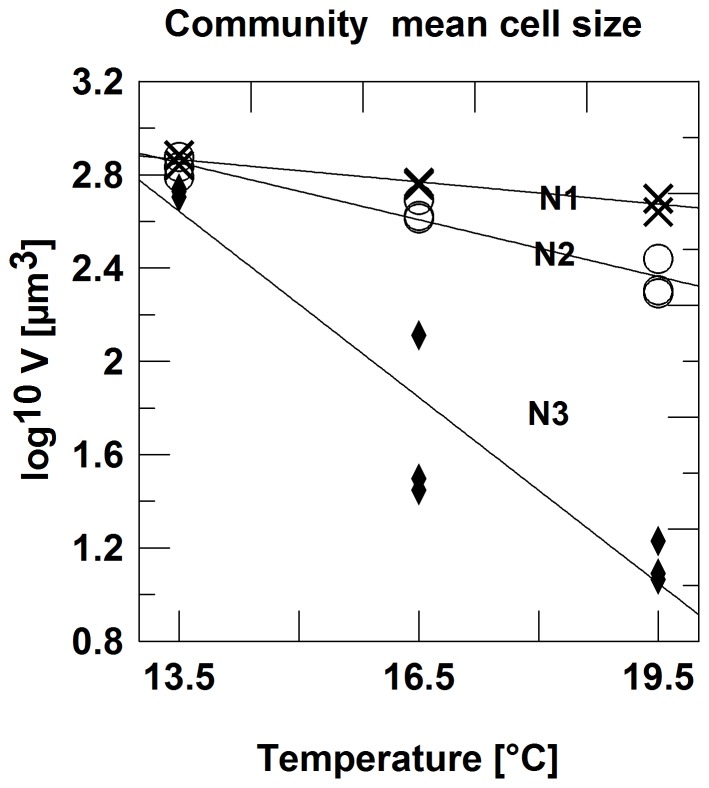
Phytoplankton community mean cell size decreases with temperature and increases with dilution rate. Community mean cell volume in µm^3^ (log^10^-scale) in response to temperature and nutrient regime; N1: 50% dilution three times per week; N2: 25% dilution three times per week; N3: no dilution.

**Figure 3:**
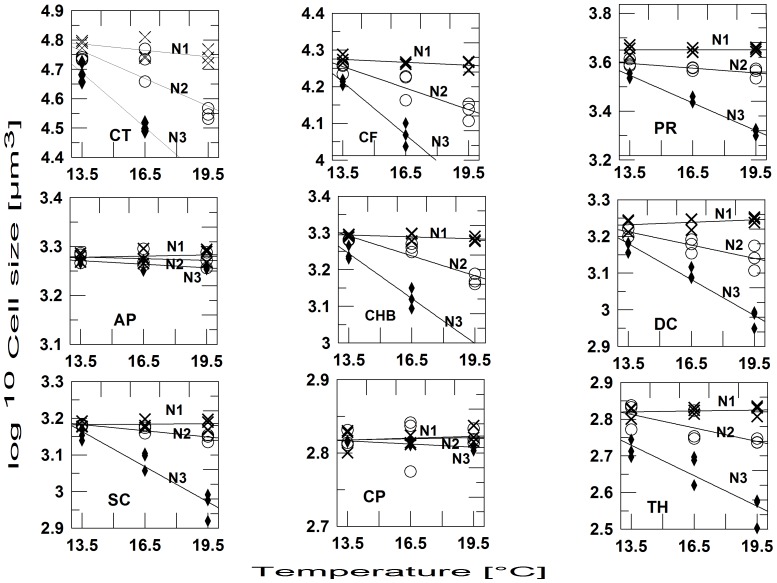
Cell sizes of phytoplankton taxa decrease with temperature and increase with dilution rate. Cell volume in µm^3^ (log^10^-scale) in response to temperature and nutrient regime; N1: 50% dilution three times per week; N2: 25% dilution three times per week; N3: no dilution. Species codes: CT: *Ceratium tripos*; CF: *Ceratium fusus*; PR: *Prorocentrum micans*; AP: *Amphidinium* sp.; CHB: *Chaetoceros brevis*; DC: *Dictyocha speculum*; SC: *Scrippsiella trochoidea*; CP: *Cerataulina pelagica*; TH: *Thalassionema nitzschioides*.

**Figure 4:**
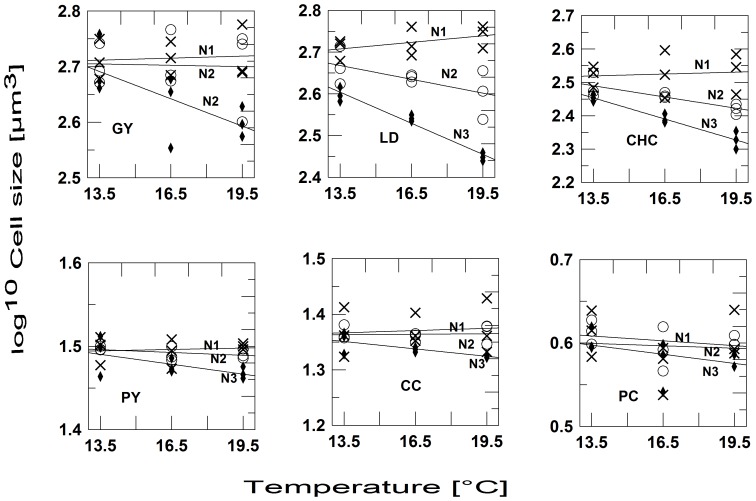
Cell sizes of phytoplankton taxa decrease with temperature and increase with dilution rate (continued). Cell volume in µm^3^ (log^10^-scale) in response to temperature and nutrient regime; N1: 50% dilution three times per week; N2: 25% dilution three times per week; N3: no dilution. Species codes: GY: *Gymnodinium* sp.; LD: *Leptocylindrus danicus*; CHC: *Chaetoceros curvisetus*; PY: *Pyramimonas* sp.; CC: *Cylindrotheca closterium*; PC: picophytoplankton.

In total, we could distinguish and count 15 species. Other protists, including heterotrophic ones were too rare to be counted. The community mean cell volume (Fig. 2) and the cell volumes of the majority of the individual species (Fig. 3, Fig. 4) showed a significant negative effect of nutrient stress (13 of 15 spp.), a negative temperature effect (10 of 15 spp.) and an interaction effect (9 of 15 spp.) (Table 1). Individual regression analyses for different levels of nutrient stress showed, that the slopes of the temperature-size relationship became more negative at more stringent nutrient stress (Table 2). At the lowest nutrient stress level, no species showed a significant response to temperature. Comparing the response of *V_c_* to the responses of *V_i_*, it is also obvious that compositional changes, i.e. dominance shifts between differently sized species, by far outweigh intraspecific size shifts. The slopes of the *Vc*-temperature regression roughly conform to ca. 4.7% size reduction per °C at N1, 17.2% at N2, and 46% at N3, respectively. The most responsive individual species, the dinoflagellate *Ceratium tripos*, decreased by 1.7% per °C at N1 (insignificant), 6.8% at N2, and 13.3% at N3, respectively.

**Table 1:** ANOVA of temperature and nutrient effects.

Species	Higher taxon	*P_temp_*	*P_nutr_*	*P_int_*	*R^2^*
Community mean cell size		<0.001	<0.001	<0.001	0.92
*Ceratium tripos*	Dinophyta	<0.001	<0.001	<0.001	0.89
*Ceratium fusus*	Dinophyta	<0.001	<0.001	<0.001	0.88
*Prorocentrum micans*	Dinophyta	<0.001	<0.001	<0.001	0.92
*Amphidinium* sp.	Dinophyta	0.65	0.03	0.74	0.37
*Chaetoceros brevis*	Bacillariophyceae	<0.001	<0.001	<0.001	0.94
*Dictyocha speculum*	Dictychophyceae	<0.001	<0.001	<0.001	0.89
*Scrippsiella trochoidea*	Dinophyta	<0.001	<0.001	0.002	0.79
*Thalassionema nitzschioides*	Bacillariophyceae	<0.001	<0.001	0.001	0.85
*Cerataulina pelagica*	Bacillariophyceae	0.93	0.49	0.92	0.12
*Gymnodinium* sp.	Dinophyta	0.42	0.01	0.4	0.24
*Leptocylindrus danicus*	Dinophyta	0.002	<0.001	0.002	0.79
*Chaetoceros curvisetus*	Bacillariophyceae	0.01	<0.001	0.07	0.73
*Pyramimonas* sp.	Prasinophycea	0.24	0.02	0.5	0.48
*Cylindrotheca closterium*	Bacillariophycea	0.8	0.03	0.8	0.35
Picophytoplankton	diverse taxa	0.04	0.3	0.7	0.15

Two-factor ANOVA of temperature and nutrient level effects on log^10^ cell volume (µm^3^) of the entire phytoplankton community and of the individual species arranged by size from the largest to the smallest; *N* = 27, except for *Ceratium tripos, Ceratium fusus*, and *Chaetoceros brevis* which disappeared from the N3 – 19.5°C treatment combination (*N* = 24).

**Table 2:** Regression analysis of temperature-size relationships.

Species	Nutrient level	*a*	*b*	*P*	*R2*
Community mean cell size	N1	−0.0207	3.0903	0.0009	0.88
	N2	−0.0820	3.9612	0.0003	0.90
	N3	−0.2661	6.2376	0.00008	0.92
*Ceratium tripos*	N1	−0.0073	4.8846	0.075	0.38
	N2	−0.0306	5.184	00038	0.72
	N3	−0.0619	5.5233	0.001	0.94
*Ceratium fusus*	N1	−0.0026	4.3100	0.08	0.35
	N2	−0.0196	4.5197	0.0008	0.81
	N3	−0.0479	4.8597	0.0016	0.93
*Prorocentrum micans*	N1	+0.0001	3.6486	0.950	0.0006
	N2	−0.0065	3.6831	0.012	0.61
	N3	−0.0377	4.0556	0.00003	0.96
*Amphidinium* sp.	N1	+0.0008	3.2672	0.6478	0.03
	N2	−0.0011	3.2941	0.5736	0.05
	N3	−0.1124	3.3042	0.04561	0.46
*Chaetoceros brevis*	N1	+0.0017	3.3174	0.1539	0.26
	N2	−0.0180	3.5354	0.0003	0.86
	N3	−0.0408	3.7946	0.0033	0.90
*Dicytocha speculum*	N1	+0.0022	3.218	0.2799	0.16
	N2	−0.0121	3.3756	0.00470	0.70
	N3	−0.0326	3.6202	0.00001	0.93
*Scrippsiella trochoidea*	N1	+0.0004	3.1765	0.78550	0.01
	N2	−0.0056	3.2590	0.0019	0.76
	N3	−0.0320	3.5964	0.0001	0.90
*Thalassionema nitzschioides*	N1	+0.0008	2.809	0.6558	0.03
	N2	−0.0123	2.979	0.0195	0.56
	N3	−0.0277	3.103	0.0012	0.80
*Cerataulina pelagica*	N1	+0.0008	2.8065	0.6178	0.04
	N2	+0.0005	2.8110	0.8644	0.005
	N3	−0.0014	2.8358	0.0113	0.62
*Gymnodinium* sp.	N1	+0.0012	2.6954	0.8142	0.09
	N2	−0.0008	2.7155	0.9204	0.04
	N3	−0.0164	2.9123	0.00439	0.68
*Leptocylindrus danicus*	N1	+0.0053	2.6367	0.20	0.22
	N2	−0.0111	2.8173	0.08	0.37
	N3	−0.0249	2.9397	0.00006	0.94
*Chaetoceros curvisetus*	N1	+0.0019	2.4940	0.8022	0.009
	N2	−0.0112	2.6416	0.0077	0.66
	N3	−0.0211	2.7378	0.0004	0.92
*Pyramimons* sp.	N1	+0.005	1.4871	0.779	0.009
	N2	−0.0011	1.5115	0.2010	0.23
	N3	−0.0040	1.5436	0.05015	0.45
*Cylindrotheca closterium*	N1	+0.0013	1.3485	0.8052	0.009
	N2	+0.0003	1.3598	0.8929	0.003
	N3	−0.0044	1.4106	0.0239	0.54
Picophytoplankton	N1	−0.0010	0.6125	0.8400	0.006
	N2	−0.0019	0.6344	0.4637	0.008
	N3	−0.0037	0.6467	0.2290	0.19

Regression (Model: y = ax+b) of log^10^ cell volume (µm^3^) on temperature (°C) for the different species and nutrient levels; *N* = 9, except for *Ceratium tripos, Ceratium fusus*, and *Chaetoceros brevis* which disappeared from the N3 – 19.5°C treatment combination (*N* = 6).

## Discussion

While the dominance of the nutrient effect in mediating the temperature-size effect is obvious, a remaining nutrient-independent role of temperature cannot be assessed from a direct comparison of the different treatments, because temperature itself influenced the strength of nutrient stress, as can be seen from the response of the C∶N ratios to temperature. However, if the C∶N ratio is taken as indicative of the intensity of nutrient stress [21], a nutrient-independent effect can be assessed by a multiple regression with temperature and C∶N-ratios as independent variables (Table 3). The dominance of the nutrient effect is obvious, both from the number of significant cases and from the partial correlation coefficients. The mean nutrient-independent temperature regression slope was −0.0059±0.0028 (95% CL). The slope for the community level effect indicates a 19.3% size reduction, while the most temperature sensitive species, *Ceratium tripos*, showed a 4.1% size reduction per °C. The mean value of species specific size reduction was 1.36% (SD = 1.16; Shapiro-Wilks test for normality: p = 0.24), while several species did not show any nutrient-independent temperature response. Overall, the range of variation overlaps with the results obtained from clonal cultures of a wide array of protists [5].

**Table 3:** Multiple Regression of cell volume on temperature and C∶N ratios.

Species	*a*	*b*	%°C^−1^	*c*	*P_t_*	*R_t_*	*P_CN_*	*R_CN_*	*P_model_*	*R^2^*
Community mean cell size	6.55	−0.093	−19.3	−2.22	0.0011	−0.40	<0.0001	−0.69	<0.0001	0.71
*Ceratium tripos*	5.55	−0.018	−4.1	−0.49	0.0019	−0.35	<0.0001	−0.55	<0.0001	0.66
*Ceratium fusus*	4.8	−0.011	−2.5	−0.36	0.005	−0.34	<0.0001	−0.54	<0.001	0.66
*Prorocentrum micans*	4.28	−0.008	−1.8	−0.51	0.045	−0.18	<0.0001	−0.86	<0.001	0.81
*Amphidinium* sp.	3.33	−0.001	−0.23	−0.04	0.67	−0.07	0.0046	−0.54	0.012	0.66
*Chaetoceros brevis*	3.76	−0.01	−2.3	−0.31	0.0038	−0.35	<0.0001	−0.54	<0.0001	0.68
*Dictyocha* sp.	3.75	−0.009	−2.1	−0.36	0.011	−0.28	<0.0001	−0.78	<0.0001	0.75
*Scrippsiella trochoidea*	3.62	−0.009	−2.1	−0.29	0.017	−0.30	<0.0001	−0.72	<0.0001	0.66
*Thassionema nitzschioides*	3.38	−0.007	−1.6	−0.44	0.024	−0.20	<0.0001	−0.86	<0.0001	0.83
*Cerataulina pelagica*	2.83	0.0002	+0.05	−0.017	0.85	+0.38	0.24	−0.23	0.54	0
*Gymnodinium* sp.	2.97	−0.003	−0.7	−0.20	0.49	−0.11	0.0006	−0.64	0.0014	0.37
*Leptocylindrus danicus*	3.23	−0.004	−0.92	−0.45	0.23	−0.11	<0.0001	−0.87	<0.0001	0.79
*Chaetoceros curvisetus*	2.93	−0.006	−1.4	−0.32	0.071	−0.20	<0.0001	−0.80	<0.0001	0.71
*Pyramimonas* sp.	1.56	−0.001	−0.23	−0.047	0.26	−0.17	0.0029	−0.50	0.0038	0.32
*Cylindrotheca closterium*	1.45	0.0002	+0.05	−0.085	0.92	+0.02	0.003	−0.57	0.0102	0.26
Pico-phytoplankton	0.65	0.002	−0.46	−0.017	0.33	−0.20	0.52	−0.13	0.43	0

Regression according to the model log^10^ V = a+b.t+c.log^10^(C∶N), where t is expressed in °C and V in µm^3C^; partial correlation coefficients (*R_t_, R_CN_*), partial probabilities of error (*P_t_, P_CN_*), *R^2^* for the full model, and probability of error for the full model (*P_model_*); *N* = 9, except for *Ceratium tripos, Ceratium fusus*, and *Chaetoceros brevis* which disappeared from the N3 – 19.5°C treatment combination (*N* = 6). The temperature effect is also shown as % volume reduction per °C.

An alternative explanation for the observed temperature effect could lie in the dilution effect on protistan grazers (microzooplankton) which are more strongly diluted at higher dilution rates. Since microzooplankton in general prefer smaller prey and thereby benefit the larger prey both inter- and intraspecifically. Therefore, the grazing and the nutrient effect on cell sized should have the same sign, i.e. smaller sized at lower dilution rates. However, there are good reasons to consider the contribution of the microzooplankton effect as relatively unimportant:

1. Microzooplankton densities were too low to count them in our phytoplankton samples, as opposed to at least 100 phytoplankton cells counted per species and sample. Therefore, grazing rates must have has little influence on the outcome of our experiment.
2. In our experiment, the nutrient effect on the cell size was generally stronger for the larger species which are outside the feeding spectrum of most microzooplankton species.
3. If the effect of microzooplankton grazing dominates the size reponse of phytoplankton, higher grazing rates at warmer temperature should lead to a positive temperature effect on cell size. This hypothesis was tested in a previous study [12] and rejected. Even under protist grazing, warming led to a shrinkage of cell size, although not as strongly as under copepod grazing.

Direct temperature effects, nutrient effects, and grazing effects as explanations for temperature dependent size trends are not mutually exclusive. However, our results strongly indicate that the direct temperature effect is much weaker than the nutrient effect. This was found both at the intra- and the interspecific level. The community effect was much stronger than the intraspecific effect, but this is no surprise, because the scope for interspecific size difference by far exceeds the scope for intraspecific ones: Size differences between species span about 9 orders of magnitude while intraspecific size changes are almost always <1 order of magnitude on a volumetric base [26]. We cannot exclude additional mechanisms such as enhanced grazing under higher temperatures. However, the effect of grazing would be less consistent, because different guild of grazer affect different parts of the phytoplankton size spectrum [27], e.g. protozoan grazer would rather suppress phytoplankton <5 or 10 µm, while copepods would rather suppress larger ones, i.e. temperature effects mediated by grazing should depend on the dominance of different grazer guilds. In a previous study [12] we found the expected stronger negative temperature effect on phytoplankton under copepod grazing, while we did not find the reversal of sign under protist grazing. This means, that a grazing independent negative temperature effect on phytoplankton must have outweighed the supposed positive effect of protist grazers. Then, we could only speculate about the possible importance of nutrient limitation, while now we have provided direct evidence for it.
